# Acknowledgement to Reviewers of *Nutrients* in 2015

**DOI:** 10.3390/nu8010061

**Published:** 2016-01-21

**Authors:** Nutrients Editorial Office

**Affiliations:** MDPI AG, Klybeckstrasse 64, CH-4057 Basel, Switzerland; nutrients@mdpi.com

The editors of *Nutrients* would like to express their sincere gratitude to the following reviewers for assessing manuscripts in 2015. 

We greatly appreciate the contribution of expert reviewers, which is crucial to the journal’s editorial decision-making process. Several steps have been taken in 2015 to thank and acknowledge reviewers. Good, timely reviews are rewarded with a discount off their next MDPI publication. By creating an account on the submission system, reviewers can access details of their past reviews, see the comments of other reviewers, and download a letter of acknowledgement for their records. In addition, MDPI has launched a collaboration with Publons, a website that seeks to publicly acknowledge reviewers on a per journal basis. This is all done, of course, within the constraints of reviewer confidentiality. Feedback from reviewers shows that most see their task as a voluntary and mostly unseen work in service to the scientific community. We are grateful to our reviewers for the contribution they make.
Aaron, Grant J.Herbert, CristanPeairs, Abigail D.Abbeddou, SouheilaHerbozo, SylviaPearce, JoAbenavoli, LudovicoHernandez, TeriPeart, Daniel J.Abidi, RazaHerr, IngridPechey, RachelAbizari, Abdul-RazakHetherington, MarionPedrón-Giner, ConsueloAbner, ErinHeyninck, KarenPeinado, Juan R.Abraham, Elizheeba C.Higuchi, MitsuruPellanda, Lucia C.Abril, MarHilmers, AngelaPeluso, IlariaAdan, AnaHimmelgreen, David A.Peña, SalvadorAddis, MariaHimotoe, TakashiPeñagaricano, FranciscoAggarwal, AnjuHines, Stephanie L.Peng, Chiung-HueiAhn, Dong UkHingle, MelaniePentieva, KristinaAkoumianakis, IoannisHira, TohruPepino, M. YaninaAl-Dujaili, EmadHjartåker, AnettePeragón, JuanAlaedini, ArminHo, Sai YinPereira, Romaiana PicadaAlessandro, MussaHo, Feng-MingPereira, DavidAlexandre-Gouabau, Marie-cécile F.Hodge, A. M.Perez-Cano, FranciscoAlexy, UteHodson, LeannePerez-Jimenez, JaraAlfonso, MiguelHoevenaar-Blom, Marieke P.Pérez-López, Faustino R.Alhazmi, AmaniHoffmann, PeterPeriasamy, MuthuAllegaert, KarelHofmann, JohannPeroni, DiegoAllen, Jason D.Holick, Michael F.Perry, V. E. A.Allen, Lindsay H.Hollis, BrucePersky, SusanAllen, KatieHollman, Peter C. H.Peterson, JuliaAller, PatricioHolmes, GeoffreyPeterson, Amie L.Alles, BenjaminHolst, Jens JuulPetrie, Trent A.Almanza-Pérez, Julio CésarHolub, BrucePfeiffer, AndreasAltieri, BarbaraHönow, RuthPiccoli, G. B.Aluko, RotimiHoriuchi, MasahisaPiccolo, Brian D.Alwan, NaHornstra, GerardPicklo, Matthew J.Alway, Stephen E.Horrigan, Louise A.Picó, CatalinaAmato, Katherine R.Hosking, JoannePietzak, MichelleAmoaku, Winfried M.Hou, Wen-ChiPifferi, FabienAnderson, Alex KojoHouston, Mark C.Pignitter, MarcAnderson, Joel G. Howatson, GlynPipingas, AndrewAnderson, GregHowes, MelaniePisciotta, LiviaAnderson, JohnHøiriis Nielsen, JensPittas, AnastassiosAndjelkovic, Anuska V.Huang, MingguoPlaza-Díaz, JulioAndrade, FernandoHuang, JosephPoblaciones, Maria J.Andrade, PaulaHuang, Chi-ChangPolesel, JerryAndreozzi, PaoloHuczyński, AdamPolidori, Maria CristinaAndres, AlineHuffman, Kelly J.Polimeni, AntonellaAnnunziata, AzzurraHughes, DavidPompei, RaffaelloAnstey, NickHughes, Christine A.Poppitt, SallyAntonogeorgos, G.Hulton, Andrew T.Poti, Jennifer M.Apolzan, JohnHurrell, RichardPotischman, NancyApostolova, NadezdaHusby, Steffen Powers, Hilary J.Appleton, KatherineHwang, Dae YounPowles, John W.Arany, EdithHyldig, GrethePrabhu, SandeepArisawa, KokichiIaniro, GianlucaPradelli, LorenzoArjmandi, Bahram H.Iannitti, TommasoPratsinis, HarrisArmand, MartineIannotti, Ronald J.Pravst, IgorArmstead, William M.Ibrahim, Salam A. Probst, YasmineArola-Arnal, AnnaIgarashi, KiharuProtiva, Petr ProtivaAselage, Melissa B.Ilich, Jasminka Z.Pufal , Milene A. Ashida, HitoshiImamura, FumiakiPuglisi-Allegra, StefanoAsuero, Agustín G.Ishihara, HisamitsuPumpa, KateAtkinson, SarahIshii, IsaoPurchas, Roger W.Atkinson, LouIshii, TakeoQi, QibinAugustin, HannaIslam, MunirulQuan, Stuart F.Aura, Anna-MarjaIvey, Kerry L.Quattrin, TeresaAuricchio, SalvatoreIzawa, TakeshiQuigley, EamonAvery, A.Jaacks, Lindsay M.Raatz, Susan K.Azcárate-Peril, M. AndreaJablonski, KristenRachek, LyudmilaAzcona, CristinaJaceldo-Siegl, KarenRadmacher, PaulaBaar, KeithJackson, PhilippaRadnitz, Cynthia L.Babu, DineshJacobs, PatrickRajasekaran, KamalakannanBae, Jong-MyonJacobson, SandraRaju, JayadevBailey, MichaelJacquot, YvesRalston, PennyBailey, ReganJaczynski, JacekRamalingam, LathaBakare, OladapoJadeja, RavirajsinhRamos, SoniaBaker, JulienJaeschke, HartmutRamos, ElisabeteBaker, Lindsay B.Jahan-Parvar, MohammadRamsay, J. RussellBakker, SjoerdJahns, LisaRamsvik, Marie Sannes Bakker, Barbara M.Jain, RakeshRanadheera, C. SenakaBammann, KarinJames, Morgan H.Ranganathan, NatarajanBanach, MaciejJanda, M.Rapoport, Stanley I.Banerjee, KalpitaJang, Byoung KukRas, Rouyanne T.Banskota, Arjun H.Jankovic, RadmilaRasmussen, Heather E.Baragetti, AndreaJansen, Tim L.Raterman, H. G. RatermanBarak, MiraJansen, EugenRazzaghi, HildaBaranowski, TomJaspers, Richard T.Reading, Benjamin J.Barbaresko, JanettJawadwala, RehanaRealdon, StefanoBarbarossa, Iole TomassiniJean-Marc Tadié, Jean-MarcRedman, Leanne M.Barbour, JayneJeanes, YvonneRees, WilliamBarceló, JuanJeejeebhoy, KhursheedRegina, Brigelius-FloheBarclay, DenisJeffery, IanReicks, MarlaBarker, TylerJenkins, TrishaReinders, JoergBarker, Felix M., IIJenkins, GarethRenaud, JustineBarnes, D. W.Jenkins, D. J. A.Renault, KristinaBarrajón-Catalán, EnriqueJenkins, ArthurRenis, MarcelaBarreau, FrederickJensen, Morten GeorgRespondek, FrederiqueBarrero, AnnaJeong, DaewonReverri, ElizabethBarreto, Maria Do CarmoJiang, QingReynolds, Clare M.Barrett, AnnJohansson, EvaRezzani, RitaBarry, Elizabeth L. R.Johnson, Jeremy J.Rhodes, JonathanBarter, Philip J.Johnson, David A.Richardson, KrisBartolomé, BegoñaJohnson, Sarah A.Riddell, LynnBasini, GiuseppinaJohnson, RichardRigano, DanielaBassareo, Pier PaoloJohnson, ElizabethRigo, JacquesBasu, ArpitaJohnson, IanRiley, Malcolm D.Battino, MaurizioJohnston, CarolRinaudo, PaoloBattistoni, AndreaJohnstone, Alexandra MRink, LotharBaum, JamieJoo, Nam-seokRivas, AnaBaum, Marianna K.Jorda, CailinRivellese, Angela A.Bay, Jacquie L.Jordon, SueRiverin, BrunoBazer, Fuller W.Joseph, JacobRizvi, Syed IbrahimBazzano, Lydia A.Joven, JorgeRizzo, Angela MariaBeasley, Jeannette M.Joy, Edward J. M.Roa Romero, Laura M.Beezhold, Bonnie L.Judd, Robert L. Robert-McComb, Jacalyn J.Behrens, JürgenJulia, ChantalRoberts, JustinBehrens, GundulaJuonala, MarkusRobertson, GrahamBei, RobertoJurewitsch, BrianRobertson, Hugh M.Bel Serrat, SilviaJurk, DianaRobertson, M. DeniseBell-Anderon, KimJuszczak, LesławRoche, M.Bellastella, GiuseppeKabbani, Toufic A.Rocic, PetraBellisle, FranceKaikkonen, JariRödel, FranzBelski, ReginaKaiyala, Karl J.Rodriguez-Casado, ArantxaBenatar, Jocelyne R.Kaji, HiroshiRodriguez-Mateos, Ana M.Bennett, LouiseKalf, J. G.Rogers, Lynette K.Bentley, DavidKam-sang, WooRohrmann, SabineBentsen, H.Kamolz, LarsRoman, Henry A.Benyshek, Daniel C.Kanaley, Jill A.Romero, AlejandroBerge, Rolf KristianKang, Bum-YongRondanelli, MariangelaBerger, JacquesKang, Jae HeeRosan Meyer, RosanBerger, KarinKanoski, Scott E.Rosborg, I.Bergman, PeterKaplan, Bonnie J.Rose, DevinBernardi, FrancescoKappen, ClaudiaRoseman, Mary G.Bernhard, WolfgangKarkhanis, VrajeshRosendale, Douglas IanBerry, Robert J.Karlic, HeidrunRosenkilde, MadsBert, BravenboerKarras, S. N.Rosinger, AsherBertino, Joseph R.Kasarda, DonRoss, A. CatherineBertolo, RobertKaser, SusanneRoss, Alastair B.Bertone-Johnson, Elizabeth R.Kastner, ThomasRossi, MauroBetts, JamesKatayama, ShigeruRostami, KamranBevilacqua, AntonioKato, HisanoriRousseau, G.Bhagwat, SeemaKatuchova, JanaRowland, NeilBhatta, AnilKatzberg, Hans DieterRubini, MicheleBiesiekierski, JessicaKaur, GunveenRucklidge, JuliaBirch, EdwardKawabata, KyuichiRudel, LawrenceBitsch, RolandKawai, YoshichikaRuden, DouglasBizzaro, NicolaKawasaki, HiromuRuderman, Neil B.Bizzaro, GiorgiaKeast, RussellRudkowska, IwonaBjornstad, PetterKeely, SimonRudzitis-Auth, JeannetteBjornstedt, MikaelKegler, MichelleRufián Henares, José ÁngelBjørndal, BodilKellow, N. J.Rumbold, PennyBlackshaw, L. AshleyKenborg, LineRune, Gabriele M.Blair, JessicaKennedy, Deborah A.Russell, Mark T.Blanton, CynthiaKenyon, ChrisRutten, Erica Blasco-baque, VincentKersting, MathildeRyan, LisaBlesso, ChristopherKharbanda, KusumRyan, ElizabethBlumberg, JeffreyKhoo, JoanRyan, DanielleBlumfield, MichelleKhosla, PramodRyckman, Kelli K.Blundell, Jonh Ki, Sung HwanRylander, RagnarBoaz, MonaKida, SatoshiSaavalainen, P.Bobe, GerdKido, YoshiakiSabbagh, Heba JafarBogl, L. H.Kiefte-De Jong, JessicaSachan, Dileep S.Bolo, NicolasKim, HailSaisho, Y.Bonaccorsi, GuglielmoKim, Jin-KyungSaito, YoshiroBondonno, CathyKim, Kwang-OkSakhaee, KhashayarBoniol, MathieuKim, YoungsooSakuma, KunihiroBonnefont-Rousselot,Kim, Nam DeukSalem, NormanDominiqueKim, JunghyunSaliba, Anthony J.Booth, SarahKim, Na-hyungSalisbury, ElisabethBoqué, NoemíKim, Chang H. Salmerón, IvanBoquien, Clair-YvesKim, HojunSalsberry, Pamela J.Bordoni, AlessandraKim, Hyun-SookSalvatore, FrancescoBorghi, ClaudioKim, JaeryongSalvini, FilippoBörnhorst, ClaudiaKim, Yoon-KeunSamocha-Bonet, DoritBorrelli, FrancescaKim, SunohSánchez, María-JoséBortolus, RenataKim, YuriSánchez-Fidalgo, SusanaBosi, PaoloKim, Jae-SungSanders, TomBouillon, RogerKim, Chang-OSanders, KerrieBozzetti, FedericoKim, Kil-SooSanders, Mary EllenBradbury, Kathryn E.Kim, Kyung-suSantilli, FrancescaBradshaw, Patrick C.Kimokoti, Ruth W. Santini, AntonelloBrain, Susan D.Kimura, KazunoriSantoro, DomenicoBrandhagen, MartinKimura, HidetoSantos, DeboraBrantsæter, Anne LiseKing, NeilSantulli, GaetanoBravi, FrancescaKingsley, MichaelSasazuki, S.Bravo, R.Kipnis, VictorSato, ShinBravo, LauraKipp, Anna P.Sato, Eisuke F.Breitenfeld Granadeiro, LuizaKirk, SaraSatokari, ReettaBrenna, J. ThomasKirkpatrick, Sharon I.Satta, P. UsaiBrennan, K. M.Kitahara, Cari Sauder, Katherine AnnBrennan, LorraineKitson, AlexSavaiano, DennisBrewer, George J.KLARIN, B.Savary-Auzeloux, IsabelleBriand, LoicKlaus, SusanneSavastano, SilviaBrisswalter, JeanickKlims-Zacas, Dorothy J.Savino, FrancescoBrites, AliceKluza, JeromeSayón-Orea, CarmenBrodribb, WendyKłęk, StanisławSazawal, SunilBrough, LouiseKneepkens, C. M. FrankSbrana, CristianaBrough, HelenKnezeic-Jugovic, ZoricaScalbert, AugustinBrouns, FredKniefel, WolfgangSchaefer, Adam M.Brown, RachelKo, Hyun-JeongSchauss, Alexander G. Brown, LindsayKo, Kam MingSchebendach, JanetBrown, Andrew WKo, Byung-JoonSchembre, Susan M.Brownlee, IainKöcher, ThomasSchengrund, Cara-lynneBruckbauer, AntjeKoehler, PeterScherag, AndreBucca, CaterinaKoenigstorfer, JoergSchippa, SerenaBucher, TamaraKohli, RohitSchlegel, AmnonBuchowski, Maciej S.Kolb, AndreasSchlesinger, SabrinaBuckland, Nicola J.Kolehmainen, MarjukkaSchlieper, GeorgBudovskaya, YelenaKolodziejczyk, JoannaSchluns, Kimberly S.Burckhardt, PeterKolsteren, PatrickSchoeller, DaleBurke, Rachel M.König, DanielSchoenaker, Danielle AJMBurrin, DouglasKonstantinidou, ValentiniSchomburg, LutzBurrows, Tracy L.Kontoyianni, MeropiSchuh, Leslie M.Buscemi, SilvioKostrzynska, MagdalenaSchulze, KerryBuyken, Anette E.Kousa, AnneSchurgers, L. J.Byberg, LiisaKoyama, YuSchwab, UrsulaByrne, Linda K.Koyama, HiroshiSchwarzer, RalfCaffarelli, CarloKoziel, HenrySchweizer, UlrichCafolla, ArturoKozyrskyj, Anita L.Schwendeman, AnnaCagnacci, AngeloKraguljac, Nina VanessaSchwentner, LukasCalabuig-Navarro, VirtuKrajka-Kużnik, ViolettaSchwingshackl, L.Calamai, MartinoKrautwurst, DietmarScott, KarenCalder, Philip C.Kreider, RichardScott, M ECaldwell, Kathleen L.Kremers, StefScott, SamCallister, RobinKroeker, Karen I.Sculley, Dean V.Calon, FrédéricKrol, EwelinaSeal, JudyCalvo, MonaKrueger, ThiloSeehaus, StefanieCamargo, CarlosKubis, Hans-PeterSeetharaman, ShyamCamfield, DavidKubota, MasaruSekiguchi, MasayukiCanbay, AliKuda, TakashiSellayah, DyanCanene-Adams, KirstieKudsk, KennethSelma, María V.Carlsen, Monica H.Kuhlenschmidt, Mark S.Selsby, JoshuaCarnés, JerónimoKuhnle, G. G. C.Sen, UtpalCarrieri, PatriziaKuntz, SabineSerra, SerraCarroccio, AntonioKuo, Shiu-MingSerra, DolorsCarson, Scott AlanKuo, LihSerra, M. C.Carter, GrahamKupetsky-Rincon, Erine A.Servais, StéphaneCasalino, ElisabettaKuraku, ShigehiroServin, Alain L.Casanovas, CarmenKurotani, KayoSeubert, John M.Castan-Laurell, IsabelleKussmann, MartinSevilla, María-ÁngelesCaudill, MarieKusuhara, HiroyukiShaheen, RubinaCengotitabengoa, MonicaKwok, Timothy C. Y.Shaikh, Saame RazaCerella, ClaudiaLacan, DominiqueSharma, Girdhari M.Chaffee, BenjaminLachaux, Alain G.Shea, KylaChamari, KarimLadberg, RikardShearer, MartinChan, Yong ShinLagarde, MichelShehata, Awad A.Chan, Lingtak-NeanderLagerkvist, Carl JohanShen, Xiang ChunChan, Marion M.Lagiou, PagonaShen, LeslieChand, SourabhLagisz, MalgorzataShen, Szu-ChuanChang, MattLahera, VicenteShertzer, Howard G.Chang, YuanshiunLai, Hsin-ChihShim, Ki-ShukChang, SuyingLaitinen, KirsiShimada, TsutomuChang, Alex R.Lallès, Jean-PaulShimoda, HiroshiChatenoud, LucienneLam, Yan Y.Shinn, SaraChaumontet, CatherineLammi-Keefe, Carol J.Shiva, SrutiChauveau, PhilippeLamuela Raventós, Rosa MaríaShoben, Abigail B.Cheetham, TimLand, Mary-AnneShoenfeld, YehudaChen, Chung-YiLandete, José MariaSholkamy, EmanChen, Zhe-ShengLange, ChristianShomaker, LaurenChen, Jin-RanLanou, Amy JoyShui, Irene M.Chen, Ling WeiLanza, Ian R.Siafarikas, ArisChen, HuiLarraza, SebastianSibley, EricChen, Jing-HsienLarsen, JesperSieber, FritzChen, BaoshengLarsen, R. N.Silano, MarcoChen, YifanLarsson, IngridSimeonov, V.Chen, ChanghanLasekan, John B.Simirgiotis, Mario J.Chen, Yung-hsiangLass, AchimSimon, E.Chen, Yuh-FungLatruffe, NorbertSimpoulos, Artemis P.Cheng, XianwuLattard, VirginieSingh, I.Cheng, H. L.Laughlin, Maren R.Singh, Udai P.Cherbuy, ClaireLauretani, FulvioSiniscalco, DarioCherniack, E. PaulLauritzen, LotteSips, Patrick Y.Cheung, Peter C. K.Laviano, AlessandroSiristatidis, CharalamposChevalley, ThierryLawson, CharlotteSirtori, CesareChilds, EmmaLayman, Donald K.Skaaby, TeaChilibeck, PhilLayman, DonaldSkalicka-Woźniak, KrystynaChirumbolo, SalvatoreLazarus, John H.Skaltsounis, LeandrosChitchumroonchokchai, JulieLe Huerou-Luron, Isabelle Skeie, GuriChiva-Blanch, G.Lebwohl, BenjaminSkrzydlewska, ElżbietaChmurzynska, AgataLeCheminant, James D.Skuladottir, Gudrun V.Cho, SusanLedoux, Tracey A.Slavin, JoanneChobot, VladimirLee, Terence Kin-WahSluimer, JudithChoi, Yung HyunLee, Jun HeeSlupsky, Carolyn M.Choi, Wan SungLee, YoungJooSmedslund, GeirChong, Mary F. F.Lee, Jeong-chaeSmith, StephenChrist, BrunoLee, ByeongSmith, Claire M.Christakos, SylviaLee, S. H.Smith, GordonChristie, CatherineLee, Ming-FenSmith, DavidChristophersen, AsbjornLee, GordonSmyth, AndrewChristophersen, Olav AlbertLee, Wen-SenSoenen, StijnChrousos, GeorgeLee, Yoon KwangSojic, AleksandraChung, Ji HyungLee, Dong GunSomerset, ShawnChyau, Charng-CherngLee, SoJungSong, Dong-KeunCialdella-Kam, LynnLee, Moon-KyuSoni, Chirag V.Cicero, Arrigo F.G.Lee, JimmySopher, Aviva B.Clark, M. K.Lee, Tzong-shyuanSousa, CarolinaClarke, Neil DavidLeech, Rebecca M.Spano, GiuseppeClarke, GerardLeem, Kang-HyunSpecker, BonnyClaudia, VetraniLegetić, BrankaSpeckmann, BodoCleary, Margot P.Lei, XiaSpedding, SimonClemens, RogerLeiherer, AndreasSpégel, PeterClere, NicolasLeitzmann, MichaelSpence, J. David Clerici, MarioLelievre, SophieSpiller, Robin C.Clifton, P. M.Lems, WillemSquitti, RosannaClifton, PeterLeng, ShuguangStadlbauer, V.Clinton, Steven K.Leopold, Jane A.Stadler, Diane D.Coad, JaneLevant, Beth Standl, MarieCochran, BlakeLevenson, C. W.Stanley, RogerCoëffier, MoïseLeventakou, VasilikiStanner, SaraCogswell, Mary E.Lever, MichaelStark, MichaelCohen, RonaldLevy, LouisStark, KenCohen, Pieter A.Li, ShanshanStea, TonjeCollado, Pilar SánchezLi, Yu-TehStefanidou, Maria E.Colleran, Heather L.Li, WeikaiStefanska, BarbaraCollier, AbbyLi, MinSteiner, Jennifer L.Collins, MarissaLi, George Q.Steinmann, PeterCollins, Michael A.Li, Ding-YouSteinsbekk, SiljeColman, RickiLi, WeijuanStevenson, EmmaComan, Maria MagdalenaLian, MinStilli, DonatellaComino, IsabelLiaset, BjørnStokes, Caroline S.Conrad, MarcusLiberato, Selma C.Stoppe, ChristianCook-Mills, Joan M.Lidon, Fernando CebolaGenannt Bonsmann,Cooke, MattLieberman, HarrisStefan StorcksdieckCooper, Jamie A.Liebl, JohannaStote, K. S.Cornish, StephenLiem, GieStrassner, CarolaCosentino, MarcoLifschitz, CarlosStreet, S. J. Couillard, Charles Lillioja, StephenStrehle, Eugen-MatthiasCozzi, RenataLim, DongwookStritzke, WernerCozzolino, MarioLimesand, SeanStuff, Janice E.Crawford, Sybil L.Lin, Wen-YuanStulnig, Thomas M.Crescenzo, RaffaellaLin, Pao-hwaStumbo, Phyllis J.Crichton, Georgina E.Lin, Shyh-HsiangSu, Chun-LiCristofalo, ElizabethLin, Jaung GengSugahara, TakuyaCroft, KevinLindfors, KatriSuh, Jun GyoCuriel, J. A.Lio, Peter A.Sui, XuemeiCurl, Cynthia L.Lionetti, ElenaSun, MinguiD'Angelo, StefaniaLitonjua, AusgustoSun, GuangD'archivio, MassimoLiu, PeggySung, Ping-Jyunda Silva, Robin P.Liu, PhilipSutton-McDowall, Melanie L.Dadaczynski, KevinLiu, JianghongSuzuki, TakuyaDahl, AnnaLiu, ZhenhuaSvegliati Baroni, GianlucaDahle, CharlotteLo, Hui-ChenSwallow, DallasDaiko, HiroyukiLo, Yi-chenSwart, Karin M. A.Dalton, CarolineLodge, JohnSwinburn, BoydDamoiseaux, Jan Lof, MarieSze, NewmanDanesi, FrancescaLohse, BarbaraSzilagyi, AndrewDaniels-Wells, Tracy R.Lokeshwar, BalakrishnaSzymlek-Gay, Ewa Danner, Unna N.Lombardo, YolandaTahul, Maria B BadiaDarcey, ValLombó, FelipeTain, You-LinDarlow, Brian A.Lonardo, AmedeoTakahashi, DaijiroDarmon, NicoleLoomes, KerryTakahisa, KanekiyoDas, UndurtiLopez-Garcia, EstherTakatori, ShingoDaugirdas, JohnLorenz, LailaTakeuchi, HiroakiDave, Kunjan R.Lorini, ChiaraTakkunen, MarkusDavis, PaulLoughead, JamesTAKO, ELADDavison, Karen M.Louie, JimmyTalukder, Jamilur R.Davy, BrendaLowe, NicolaTan, S-Y.de Godoy, MariaLu, WenhuaTanaka, Makotode Keyzer, WillemLucan, Sean C.Tanaka, Shigeo M.de la Barca, Ana M. CalderónLucendo, Alfredo J.Tang, Lide la Monte, SuzanneLuyckx, Valerie A.Tangari, Andreade Leon, MarinoLyon, GrahamTangney, Christy C.de Magistris, LauraLyons, Timothy J.Tannen, Antjede Medina, F SánchezLyons, Paul E.Tanumihardjo, Sherryde Santi, MauroMa, Jin YeulTanus, José Eduardode Sotomayor, Maria AlvarezMa, YongjieTappia, Paramjit S.de Souza, RussellMabbott, NeilTappy, Lucde Souza, Vanessa RiosMachida, MasashiTarantino, Giovannide Tena, Jaime GarcíaMacMillan, FreyaTarasuk, Valeriede Vries, JeanneMacpherson, HelenTatti, Patriziode Young, Kyle P.Madej, DawidTavridou, AnnaDegens, HansMager, DianaTaylor, Julia A.deGraffenried, Linda A.Maggirwar, SanjayTaylor, CarlaDehghan, MahshidMaher, PamelaTaylor, Natalie Deighton, KevinMaier, Jeanette A.m.Taylor, RachelDekker, MarloesMaione, FrancescoTchernof, AndréDel Pino, JavierMakharia, GovindTe Morenga, LisaDeldicque, LouiseMakino, AyakoTeasdale, ScottDeLeon-Pennell, Kristine Y.Makse, Hernán A.Teh, Sue-SiangDelisle, HélèneMalaguarnera, MicheleTekle, M.Dellavalle, Diane M.Mallard, Simonette R.Temple, Norman J.Delvin, Edgard E.Manary, MarkTeodoro, JoãoDeminice, RafaelMancuso, CesareTeodoro, Anderson JungerDeramaudt, T. B.Mangge, HaraldTepper, Beverly J.Desai, Kaushik M.Mani, AryaThavarajah, DilDesiree Tande, DesireeMännistö, TuijaTheil, ElizabethDessìAngelica, AngelicaManor, DannyTheodora, PsaltopoulouDetzel, PatrickMantovani, GiovanniThèvenod, FrankDeutz, Nicolaas E. P.Manzanares, WilliamThiele, DoriaDharmarajan, ArunasalamMarakis, GeorgiosThiriet, MarcDharmasena, SenarathMarconi, Anna MariaThomsen, Simon FrancisDi Gioia, DianaMaréchal, EricThomson, Rebecca L.Diana, PatriziaMarfaritis, MariosThota, ChandrasekharDiciccio, PierluigialdoMaria José, Rodriguez LagunasThunders, MichelleDickey, JenniferMaria Van Rompay, MariaThunnissen, ErikDietrich-Muszalska, AnnaMarineli, RafaelaTian, JideDildy, Gary A.Mariotti, FrançoisTiboni, Gian MarioDimmock, JamesMarkofski, Melissa M.Tie, Jian-KeDimova, ElitsaMarques, ElizaTimmerman, Kyle L.Dipnall, Joanna F.Marriott, Bernadette P.Tinker, Sarah C.Dolinsky, Vernon W.Mars, MonicaTintle, NathanDomingues, FernandaMartel, FátimaTipoe, GeorgeDominguez-Salas, PaulaMartin, François-Pierre J.Tobias, DeirdreDonadio, CarloMartin, J.Tolstrup, Janne SchurmannDonin, AngelaMartin, Luc J.Tomata, YasutakeDonne, BernardMartín-Cordero, CarmenTomofuji, TakaakiDoom, Jenalee Martín-Orúe, Susana M.Tomozawa, HiroshiDorea, JoseMartineau, AdrianTong, LingyingDost, TurhanMartinez, Jesse D.Torres, SusanDragsted, LarsMartinez, InesTortora, R.Drenowatz, ClemensMartinez-Gonzalez, MigueTosi, PaolaDrew, Janice E.Maruyama, KeiTovoli, FrancescoDrougard, AnneMarventano, StefanoToxqui, LauraDrummond, Micah J. Marverti, GaetanoTozzoli, RenatoDuarte, T. L.Marzban, LucyTrabace, LuigiaDuarte-Salles, TalitaMarzocco, StefaniaTran, BichDuda-Chodak, AleksandraMaslin, KateTreesukosol, YadaDugas, LaraMaslova, EkaterinaTremblay, AngeloDugo, GiacomoMasulli, MariaTremellen, KeltonDumler, F.Mateu-de Antonio, JavierTriantafillidis, John K.Dunbar, Gary L.Matias, CatarinaTrico, DomenicoDunn, WilliamMatsui, ToshiroTripolt, NorbertDuntas, LeonidasMatsumoto, IppeiTriunfo, StefaniaDupont, JoëlleMatsumoto, KenjiTrovato, GuglielmoDurlik, MagdalenaMaukonen, JohannaTrovato, Francesca MariaDusso, AdrianaMauricio, D.Tsabouri, SophiaDutkowski, GregMauriello, GianluigiTsai, Ying ChiehDwyer, JohannaMayen, Ana-LuciaTsiplakou, Eleni Dyson, PamelaMayne, ChristopherTsirogianni, AlexandraEfird, JimmyMcauliffe, FionnualaTsuji, PetraEgi, MoritokiMcCann, AdrianTsuruya, KazuhikoEhret, Georg B.McClain, DonTucker, Larry A.Eichler, KlausMcclung, AnnaTucker, Jared M.Eilertsen, Karl-ErikMcCrickerd, KeriTurner, Russell T.Einöther, Suzanne J. L.Mcgill, Anne-TheaTurpin, SarahEl-Seedi, Hesham R.McKenna, Malachi J.Tussing-Humphreys, LisaEllinger, SabineMcLean, RachaelTwells, LaurieElliott, Michael B.McLeod, G.Tylavsky, Frances A.Ellwood, PhilippaMcLeod, KatherineTziomalos, KonstantinosElmore, Lauren CaitlinMclntosh, MichaelUnick, Jessica L.England, ClareMcmahon, EmmaUnno, KeikoEngström, NiklasMcMurray, FionaUribarri, JaimeErdman, John W.McNally, DayreUusitupa, MattiErkkilä, Arja T.McNulty, BreigeVaccari, MonicaErridge, ClettMcsorley, EmeirValentao, PatríciaEscanero, FernandoMeeusen, RomainValenti, PieraEsposito, CiroMegson, IanValentinuzz, FabioEstruch, RamonMehta, AshishValenzuela, RodrigoEvans, David C.Mehta, Jawahar L.Vallgårda, SignildEvans, E WhitneyMelamed, Michal L.van Baak, Marleen A.Eyles, Darryl W.Meltzer, Helle Margretevan de Heijning, Bert J. M.Eyles, HelenMemon, M. A.van der Poorten, DavidFabroni, SimonaMenkhorst, Ellenvan der Voet, GijsbertFairweather-Tait, SusanMessina, Louis M.van Dillen, Sonja M.E.Falasca, MarcoMeyre, Davidvan Gaal, Luc F.Fan, Hueng-ChuenMian, Caterinavan Greevenbroek, M. M. J.Fang, JimMichaelsen, Kim F.van Horn, WadeFanti, PaoloMichaud, Dominiquevan Lippevelde, WendyFarah, V.Michels, Alexander J.van Loon, Luc JcFardet, AnthonyMicke, Olivervan Muijen, P.Fares, ClaraMielenz, Manfred van Rossem, LenieFarhat, GraceMiettinen, Maijavan Schothorst, Evert M.Favier, FrançoisMigliaccio, SilviaVanselow, JensFeás, XesúsMiguel, MartaVaquero, M. PilarFelder, Robin A.Miles-Chan, Jennifer L.Vardhanabhuti, BongkoshFeng, LeiMillán Calenti, José C.Vargas, JorgeFernández-Bañares, FernandoMiller, Leland V.Varney, Veronica A.Ferreira, GuillaumeMiller, JessicaVashist, Yogesh K.Ferrie, SuzieMiller, Anthony B.Vasson, Marie-PauleField, CatherineMiller, Tracie L.Vatanparast, HassanaliFields, DavidMinden, AudreyVaughan, Roger A.Fisher, Rachel A.Miquel, AdroverVeasey, R. C.Fisher, Ffolliott M.Miranda, Jose M.Venkatesha, ShivaprasadFiszman, SusanaMirilas, PetrosVenkitanarayanan, KumarFitton, HelenMishur, Robert J.Venn, BernardFleenor, Bradley S.Misner, ScottieVerardo, VitoFlint, Harry J.Misselwitz, BenjaminVerhagen, HansFond, GuillaumeMitrou, PanayiotaVerhoeven, Adrie J.Fontecha, J.Miyairi, MayaVernoux, Jean-PaulFord, DianneMiyashita, KazuoVeronese, NicolaFord, AndrewMoazzami, AliVersen-Höync, F. vonFord, Rodney Philip KinvigMoffat, TinaVeugelers, PaulFord, StephenMohajeri, M. HasanVia, Michael A.Forde, CiaranMohammad, Ramzi M.Viernstein, HelmutFort, PatriceMoley, Kelle H.Vieux, FlorentFoskett, AndrewMonk, JenniferVignini, AriannaFoster, Jane A.Monlezun, DominiqueVillar-Taibo, R.Foster, MeikaMoore, Latetia V.Villegas, RaquelFoster, CharlesMoore, Linda W.Vinson, JoeFox, M.Moosmann, BerndVioque, J.Fragkos, KonstantinosMorabito, AntoninoVitaglione, PaolaFranchini, BelaMorán, José M.Vitetta, LuisFrank, Gail C.Moreno, J. J.Vitturi, DarioFranz, MarionMoreno, MariaVogel, UllaFranzese, AdrianaMoretti, DiegoVolta, UmbertoFraser, KarlMorris, Howard A.von Hurst, P. R.Fredrik Fernqvist, FredrikMorrison, Douglasvon Lintig, JohannesFreedman, MarjorieMorrison, Janna L.von Schacky, ClemensFreeman, Hugh JamesMorrison, Christophervon Vietinghoff, SibylleFreeman, Michael D.Moscow, Jeffrey A.Voortman, TrudyFreeman, Linnea R.Movassat, JamilehVormann, JürgenFrei, BalzMozurkewich, Ellen L.Vos, Miriam B.Freije, José M. P. Muhlhausler, BeverlyWada, RoyFreisling, HeinzMuir, JaneWagner, CarolFrench, Samuel W.Mulder, ChrisWai, Au-Yeung Kathy KaFriedman, Jacob E.Mulligan, Angela A.Wakusawa, ShinyaFriel, James K.Mullin, James M.Walker, Christa L. FischerFrøyland, LivarMulvihill, ErinWalker, M. M.Fuchs, DietmarMunir, Kashif M.Walker, Jacqueline L.Fujimori, KoMurakami, KentaroWalzem, RosemaryFujimura, TakashiMurphy, Michelle M.Wan, Jian-boFukazawa, MasanoriMurray, Andrew Wang, TaoFukuda, MitsuruMurray, JosephWang, DongFukui, MichiakiMurtaugh, Maureen A.Wang, Chia-LiangFuller, NickMuskiet, Frits A. J. Wang, JiaweiFuller-Tyszkiewicz, MatthewMutti, ElenaWang, TingFunato, HiromasaMysona, B.Wang, Tsung-ShingFuntikova, AnnaNadeau, Joseph H.Wang, YanwenFusaro, MariaNagaoka, SatoshiWard, Loren S.Gabrielsson, BrittNaito, YujiWaterhous, Therese S.Gagnon, Dominique D.Naomichi, NISHIMURAWatkins, Adam J.Gallant, Kathleen HillNash, Sarah H.Wauben, M. H. MarcaGalli, FrancescoNaska, AndronikiWeaver, ConnieGallicano, G. IanNavarra, MicheleWebb, Andrew J. Gallo, SinaNavarro, Carlos Javier GonzálezWebb, R. ClintonGalloway, Stuart D. R.Navas-Carretero, SantiagoWei, XierongGanglmair-Wooliscroft,Nazzaro, FilomenaWeijs, Teus J.Alexandra Newnham, EvanWeishaar, TomGarcia, CyrilNexo, EbbaWeiss, ScottGarcia-Larsen, VanessaNey, Denise M.Welch, Robert W.Gard, PaulNeymotin, FlorenceWeldon, Douglas A.Garden, FrancesNeyraud, EricWelham, Simon J. M. Gardner, DavidNgo, SherryWelsh, JeanGarg, Parveen K.Nguyen, Douglas L.Wendell, Stacy GelhausGarg, ManoharNguyen, PhuongWeng, Ching-FengGarland, CedricNichols, E. K.Wertz, PhilipGarrote, Jose AntonioNichols, Buford L.Westerterp, Klaas R.Gea, AlfredoNicklau, SophieWeylandt, Karsten H.Geirsdottir, MargretNicol, ChristopherWherry, Sarah JoGekle, MichaelNicoll, RachelWhisner, Corrie M.Gerbault, PascaleNieman, David C.White, Kenneth E.Ghaddar, Suad F.Nimptsch, KatharinaWhite, LouiseGiampiei, FrancescaNishikawa, YoshikazuWhitham, Dana L.Giannì, Maria LorellaNishina, HiroshiWhiting, Susan J.Gianotti, LucaNkeng-Efouet, Pepin AlangoWidhalm, KurtGiapros, VasileiosNobile, VincenzoWilcox, Matthew D.Gibson, Peter R.Noble, NatashaWiley, AndreaGibson, A. A.Nohr, D.Williams, Nancy I.Giera, MartinNolan, Laurence J.Williams, KristinaGlahn, RayNorström, FredrikWilliamson, PatriciaGlanzman, Marianne M.Norval, MaryWilliamson, DavidGlurich, IngridNørskov, Natalja P.Willoughby, DarrynGomaraschi, MonicaNugent, AnneWilson, GabrielGondalia, ShakuntlaNwosu, Benjamin UdokaWinters, CarenaGonzalez, JavierO'Connor, LauraWirt, TamaraGonzález, Sonia TiradoO'Connor, EibhlísWirth, StefanGonzalez-Alvarez, JuliaO'Neil, CarolWłodarek, DariuszGonzalez-Sanchez, EsterO'Neill, KristieWolff, DanielGoodall, StuartO’Grady, Thomas J.Wong, SamfordGoran, Michael I.O’Mahony, L.Wong, Julia MWGorczynski, PaulOates, LizaWongsiriroj, NuttapornGordeladze, Jan OxholmOberhelman, SaraWoo, Jessica GrausGorham, EdwardObi, YoshitsuguWood, CharlesGorman, Gregory S.Obrenovich, Mark E.Worm, MargittaGorman, ShelleyOchoa, JuanWright, Nicole C.Gosby, Alison K.Ocké, MargaWright, Melecia J.Gostner, Johanna M.Oelkrug, ChristopherWu, Jian-PingGower, BarbaraOgata, MakikoWu, HuaizhuGrabrucker, Andreas M.Ogra, YasumitsuWu, Chia-ChaoGracy, Robert W.Ohla, KathrinWu, Ray-changGraham, Dan J.Ohta, YoshijiWu, C. H.Granic, AntonetaOkello, EdwardWu, Chi-ReiGrant, WiliamOlfert, MelissaWycherley, Thomas P.Grant, RyanOliveira, M. Beatriz P. P.Wyse, RebeccaGrases, FelixOliviera, PauloXi, LinGreatwood, H. C.Olmedilla-Alonso, BegoñaXia, MinGreco, LuigiOlszewski, Pawel K.Xiong, YeGreen, CharlotteOrio, FrancescoYadav, Vijay K.Green, J. T.Ormsbee, MichaelYamaki, KojiGreenway, Frank L.Osakabe, NaomiYamamoto, HironoriGreer, Frank R.Osanai, T.Yamamoto, NorioGrider, ArthurOsmancevic, AmraYamanushi, Tomoko T.Grieger, JessicaOstlund, Sean B.Yang, C. S.Griffioen, MariekeOude Griep, LindaYang, Wei-hsiungGrill, EvaOzawa, YokoYang, Mhan-PyoGrippo, Paul J.Øverby, Nina C.Yang, Chung S.Groetch, MarionØyen, JannikeYannicelli, StevenGrose, Julianne H.Pabalan, Noel AYano, ShozoGrossini, Dott. E.Pacetti, DeboraYasuda, KazukiGrosso, ClaraPachon, HelenaYazdy, Mahsa M.Grosso, GiuseppePacifico, SeverinaYde, Christian ClementGuallar, PilarPacilli, MaurizioYee, Jennifer K.Guardamagna, O.Paddon-jones, DouglasYeh, Wei-LanGueant, Jean-LouisPagano, Mario AngeloYeh, Sung-LingGuglielmetti, SimonePagonopoulou, OlgaYeung, EdwinaGunnarsdottir, IngibjörgPal, LubnaYing, DanyangGupte, AnishaPalitti, FabrizioYokoyama, YoshihitoGutiérrez-Uribe, Janet A.Palmer, CaroleYorek, Mark A.Ha, TaeyeolPalmer, Amanda C.Yoshimura, YuichiHaase, HajoPan, MinhsiungYoshinaga, JunHaboubi, NadimPan, TaoYoung, Jette F.Hafeez, Bilal BinPanagiotakos, Demosthenes B.Young, WayneHafekost, KatherinePanahi, ShirinYoung, HayleyHaley, ScottPanaro, Maria AntoniettaYuan, ChangzhengHalloran, Bernard P.Pao, Li-HengYue, JunmingHambidge, Michael Paoli, PierYun, Jong WonHamden, KhaledPaoli, AntonioZablotska, L. B.Hammersley, RichardPapadaki, AngelikiZaborskis, ApolinarasHammoud, Ahmad O.Papadakis, SophiaZambelis, ThomasHanevold, Coral D.Papadimitriuou, KonstantinosZamora, RaulHankey, C. R.Papadopoulou, EleniZamora-Ros, RaulHannibal, LucianaPapetti, AdeleZanetti, MichelaHansen, Anita L.Parhofer, Klaus G.Zangara, AndreaHaque, AzizulPark, KyongZara, VincenzoHara, HiroshiPark, Mi-jungZarrelli, ArmandoHardy, SheilaPark, Kyong SooZazpe, ItziarHaring, BernhardPark, Sung-GyooZello, Gordon A.Harris, PhilipPark, YongsoonZhang, MengliangHartmann, ChristinaParker, Leslie A.Zhang, DeqiangHasegawa, HajimeParletta, NatalieZhao, LiangHavens, Peter L.Parnell, Jill A.Zheng, JoleneHayes, JohnParnell, WinsomeZhu, LixinHayes, MariaParthasarathy, SampathZiegenfuss, TimHayes, Kenneth CPase, Matthew P.Zimmermann, Michael B.Hazell, Alan S.Pasqui, FrancescaZingg, Jean-MarcHeber, DavidPassi, AlbertoZittermann, ArminHeinen, MirjamPatel, AlokaZoetendal, ErwinHellhammer, JulianePatel, Ravi MangalZosky, Graeme R.Helm, MarkPatole, SanjayZulueta, AidaHemila, HarriPavan, BarbaraZuniga, KrystleHendrich, SuzannePavlov, T. S.
Henriques, Marta H. F.Payan-Carreira, Rita


